# Factors Related to Nurse Satisfaction with Supervisor Leadership

**DOI:** 10.3390/ijerph20053933

**Published:** 2023-02-22

**Authors:** Wen-Pei Chang, Chia-Hui Wang

**Affiliations:** 1 Department of Nursing, Shuang Ho Hospital, Taipei Medical University, New Taipei City 235041, Taiwan; 2 School of Nursing, College of Nursing, Taipei Medical University, Taipei 110301, Taiwan

**Keywords:** nurse, satisfaction, supervisor, leadership, structural equation modeling

## Abstract

The satisfaction of nurses with the leadership of their supervisors exerts a positive association with their job satisfaction. This study identified factors associated with nurse satisfaction with supervisor leadership and developed a model of causal relationships based on social exchange theory. A satisfaction scale was developed to measure how nurses felt about the leadership of their supervisor, which also assessed the validity and reliability using a cross-sectional descriptive survey questionnaire administered to nurses working in a teaching hospital in northern Taiwan. A total of 607 valid questionnaires were returned. Structural equation modeling was used to test the theoretical model of this study. Only questions that were scored above 3 were included in the scale. A total of 30 questions were placed under seven constructs of this scale upon the assessment of content validity. The results indicate that satisfaction with shift schedules, educational training, and internal communication exerted direct, significant, and positive associations with satisfaction with the supervisor leadership. Furthermore, satisfaction with policies and guidelines exerted direct, significant, and positive associations with satisfaction with internal communication and exerted indirect associations with satisfaction with supervisor leadership through internal communication. In particular, satisfaction with shift schedules and internal communication were most significantly associated with satisfaction with supervisor leadership. The results of this study offer a reference for hospital management and emphasize focusing on the arrangement of nurse shifts in all departments. The establishment of diverse communication channels can enhance the degree of nurse satisfaction with supervisor leadership.

## 1. Introduction

To achieve continuous growth and development, the main goal of hospitals must be to ensure that the nursing personnel are willing to optimally execute their duties [1]. The degree of nurses’ satisfaction with the leadership of their supervisors influences the retention of nurses; thus, this relationship is a concern [2]. Zhang et al. surveyed the job satisfaction of nurses at 181 hospitals and found that 45% of the nurses were dissatisfied and that 5% intended to quit [3]. Research has also shown that satisfaction with one’s supervisor is a crucial factor impacting the job satisfaction of nurses [4]. The atmosphere at a workplace varies with the leadership style of the supervisors and is also a key factor influencing the job satisfaction of nurses [5]. Thus, if nurse supervisors can better understand the needs of nurses and to what they attach importance, it should improve the work quality of nurses and increase the retention rates, thereby creating mutual benefits and win–win situations.

Supervisors serve as the communication channel between hospitals and clinical nurses, so they must have clear work objectives and guidelines in order to communicate well with their subordinates and clarify duties [6]. Formulating good workplace policies is necessary to create people-oriented work environments, promote the health of nurses, and establish a good workplace atmosphere [7]. Implementing effective policies such as simplifying shift handovers may likely increase the nurses’ job satisfaction [8]. In nursing, educational training can develop meaningful learning relationships and can be organized in various venues and on topics such as clinical practice, education, management, and research [9]. Owens indicated that educational training provides organizations with many positive rewards such as increasing employee job satisfaction and reducing turnover intention. For this reason, supervisors should not cut educational training budgets [10]. Each type of educational training has a different impact on job satisfaction; nevertheless, any type of educational training can help to increase the satisfaction of employees with their supervisors and their work [11]. Incorporating empirical treatment into clinical educational training could help to enhance the nurses‘ competencies in empirical treatment and boost their interest and confidence in clinical work [12]. Furthermore, Kurniawaty et al. examined the influences of the work environment, stress, and job satisfaction on the turnover intention, and their results showed that the work environment had a significant and positive impact on job satisfaction, which in turn increased employee trust in the supervisors [13]. A multi-country and multilevel study reported that improved work environments and flexible shift schedules increase nurse retention as well as the nurses’ trust and satisfaction with supervisor leadership [14]. That is, working too many weekends, too little time off from work, and inflexible shift schedules may all affect the nurses’ satisfaction with the supervisor leadership [15]. Rudasingwa and Uwizeye believed that an incentive-based salary system would help to compensate employees for their outstanding performance and contributions, which ensures that employees feel fairly treated and respected [16]. Jankelová and Joniaková suggested that nurse leaders should open up communication channels, demonstrate respect and care, and always be eager to share their ideas and insights, thereby creating a more engaged workplace [17].

Social exchange theory can explain the relationships between clinical nurses and their supervisors. This theory emphasizes that interpersonal interactions are a process during which both parties display spontaneous behaviors based on engagements and beliefs and take part in the exchange of valuable resources [18]. Given that employees have a close relationship with management, employees naturally work hard to feel valued and to earn a raise, just as social exchange theory suggests. However, hard-working employees who are scolded or mistreated by their supervisors will be less motivated and make less effort to ensure fairness in social exchange [18,19]. Understanding factors that relate to satisfaction with supervisor leadership from the perspective of nurses is crucial for supervisors. At present, the tool most commonly used to gauge the job satisfaction of nurses is the Minnesota Satisfaction Questionnaire (MSQ); however, this scale was not designed with the content and uniqueness of nursing work in mind, and does not investigate the contents of supervisor leadership [20,21,22]. Although the Practice Environment Scale of Nursing Work Index (PES-NWI) targets the job satisfaction of nurses, differences in the medical environment and national culture can result in changes in the constructs of the scale [23,24]. The McCloskey/Mueller Satisfaction Scale (MMSS) has been translated into Mandarin Chinese; however, the constructs are distinctly different from those of the original scale [25].

**Figure 1:**
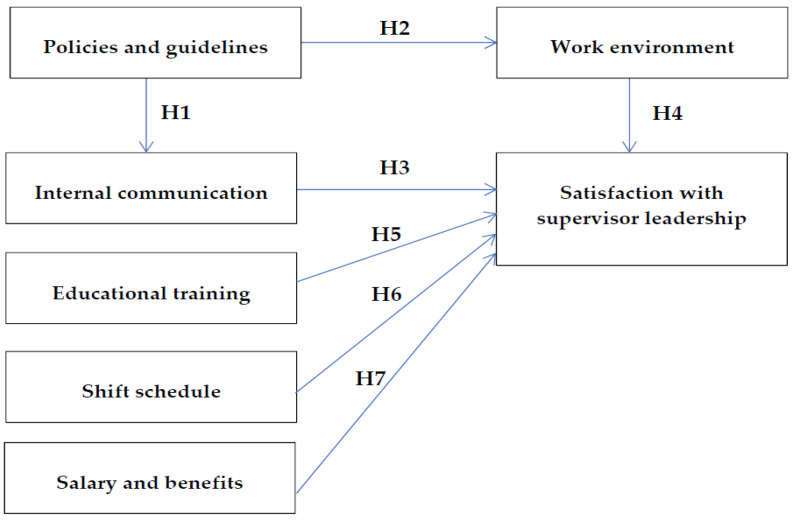
Hypothesized models of this study.

This study provided the main constructs and identified the relationships among different constructs using social exchange theory [17] by which to develop a scale that measures the nurses‘ satisfaction with supervisor leadership and assesses the validity and reliability, which ultimately identifies the relevant factors that are related to the nurses‘ satisfaction with supervisor leadership. We reviewed the relevant literature and identified the factors promoting nurse satisfaction with supervisor leadership. Thus, we developed the Nurse Satisfaction with Supervisor Leadership Scale to identify the factors that relate to the satisfaction of clinical nurses with their supervisors and attempted to construct a causal relationship model using linear structural equation modeling (SEM). Accordingly, we established the conceptual framework of this study, as illustrated in Figure 1. 

Based on this framework, we formulated the following hypotheses:

**Hypothesis 1 (H1).**  *A positive correlation exists between satisfaction with policies and guidelines and satisfaction with internal communication.*

**Hypothesis 2 (H2).**  *A positive correlation exists between satisfaction with policies and guidelines and satisfaction with the work environment.*

**Hypothesis 3 (H4).**  *A positive correlation exists between satisfaction with internal communication and satisfaction with supervisor leadership.*

**Hypothesis 4 (H4).**  *A positive correlation exists between satisfaction with the work environment and satisfaction with supervisor leadership.*

**Hypothesis 5 (H6).**  *A positive correlation exists between satisfaction with educational training and satisfaction with supervisor leadership.*

**Hypothesis 6 (H6).**  *A positive correlation exists between satisfaction with shift schedules and satisfaction with supervisor leadership.*

**Hypothesis 7 (H7).**  *A positive correlation exists between satisfaction with salary and benefits and satisfaction with supervisor leadership.*

## 2. Materials and Methods

### 2.1. Participants and Sampling 

Using purposeful sampling, this study surveyed clinical nurses, regardless of their gender, working in a teaching hospital in northern Taiwan during the period between 2 August 2017 and 1 August 2018. This hospital is affiliated with the Taipei Medical University in Taiwan. Founded in 2008, it had over a thousand beds by 2018. It focuses on acute and severe medical conditions and has emergency departments, operating rooms, acute wards in various departments, ICU, a psychiatric day care, a nursing home, and a postpartum care center. A total of 1118 nurses work in the nursing department, and they have completed pre-service and on-the-job educational training courses. Deputy head nurses, head nurses, nursing supervisors, directors, and deputy directors were excluded. We administered a questionnaire to 759 nurses. They were recruited via email, in which we guaranteed the potential respondents that this study was entirely voluntary and that they should not feel coerced or pressured to participate. We assured them that their participation, or lack thereof, would have no impact on their performance appraisal and that we would not discuss the study with potential respondents at their workplace to prevent their supervisors, who are in a position of power, from pressuring or mistreating their subordinates. The questionnaire was distributed to the nurses through email, and the completed questionnaires were returned through an electronic format anonymously. Specifically, all RNs were provided the survey via email. A total of 607 valid questionnaires were returned, yielding a valid recovery rate of 79.97%.

### 2.2. Ethics Approval and Consent to Participate 

The study was approved by our research department’s institutional review board (Taipei Medical University-Joint Institutional Review Board, project no. N201706062) before data collection. The Taipei Medical University-Joint Institutional Review Board approved a request to waive documentation for informed consent. As this hospital is affiliated with Taipei Medical University, this study had to be approved by both the IRB and Taipei Medical University before participants could be recruited. As this was not an interventional or observational study involving human biological samples, no written informed consent was needed from the staff. The subsequent statistical analyses were conducted anonymously. Furthermore, all information that could identify the staff and the collected data were encoded to protect the rights and privacy of the staff. 

### 2.3. Scale Development

#### 2.3.1. Forming Questions

This scale was designed in a way that reflected the above-mentioned literature and the practical experience of the clinical nurses in this survey. The questionnaire comprised two parts: personal information (including gender, age, educational background, and work experience) and satisfaction. The satisfaction part contained seven major constructs (Supplementary Materials File S1). These constructs examined the satisfaction of the nurses with regard to the policies and guidelines set by their supervisors (five items), their educational training arrangements (four items), their shift schedules (three items), internal communication (five items), their salary and benefits (four items), their work environment (four items), and the overall leadership of their supervisor (five items). The items were measured using a 5-point Likert scale with the following anchors: satisfaction (5), slight satisfaction (4), neutral opinion (3), slight dissatisfaction (2), and dissatisfaction (1). A higher score was considered to indicate higher nurse satisfaction with supervisor leadership.

#### 2.3.2. Expert Assessment of Content Validity 

The first draft of this scale contains 32 question items. We first had six experts assess the accuracy, appropriateness, and understandability of the content of each question item on a five-point scale (1 point indicating strong disagreement and 5 points indicating strong agreement) to obtain the item-level content validity index and the scale-level content validity index. The former equals the number of expert assessments of 3 points or higher divided by the total number of experts, and the latter equals the number of expert assessments of 3 points or higher divided by the total number of items. Index values greater than 0.8 indicated good validity; index values between 0.6 and 0.8 indicated passable validity, meaning that appropriate revisions are recommended, and it is recommended that question items with index values less than 0.6 are eliminated [24]. Following expert assessment, the revised scale contained seven constructs and 30 items. The item-level content validity index values ranged from 0.8 to 1.0, and the scale-level content validity index values ranged from 0.7 to 1.0.

#### 2.3.3. Assessing Scale Validity 

Before testing the goodness-of-fit of the overall model, we investigated offending estimates to determine whether any coefficients exceeded the acceptable range. The results revealed no negative variance in any of the error terms, all of which reached significance (*p* < 0.001). This result means that no offending estimates existed in the model; therefore, we proceeded to subsequent analyses. We next determined the goodness-of-fit by using the maximum likelihood. As shown in Table 1, most of the indices showed a good fit, demonstrating the suitability of this model for the subsequent validity assessment. The goodness-of-fit indices showed that the final model reasonably fit the data (Table 1).

We analyzed the research framework model by using confirmatory factor analysis. The standardized factor loadings of the manifest variables and their corresponding latent variables ranged from 0.67 to 0.95 (Table 2). The reliability (*R*^2^) of the individual manifest variables was between 0.443 and 0.899, and the construct reliability of the latent variables ranged from 0.87 to 0.96, thus exceeding 0.70 [26]. The average variance extracted values of the latent variables fell between 0.69 and 0.83, thus exceeding 0.50, indicating that all estimated parameters of our model met the goodness-of-fit standards [27] (Table 2). In conclusion, the questionnaire constructs showed good convergent validity.

#### 2.3.4. Assessing Scale Reliability 

IBM SPSS Amos 22.0 indicated that the Cronbach’s α values of the various constructs in our study ranged from 0.851 to 0.961; the overall Cronbach’s α was 0.936. 

### 2.4. Statistical Methods

To verify the overall theoretical model, we used IBM SPSS Amos 22.0 (United States) to obtain the descriptive statistics of the nurses and understand their demographic distributions. We then used *t*-tests and one-way ANOVAs to compare the personal characteristics of the nurses and the differences in the scores of the latent variables of the questionnaire. We subsequently examined the causal relationships among the various constructs of the model through SEM. 

## 3. Results 

### 3.1. Comparison of Nurse Personal Characteristics and Scores of the Latent Variables in the Questionnaire 

Of the 607 nurses in our study, most were women (*n* = 590, 97.2%). As the hospital has only been founded for a decade, nurses aged 20–29 years (*n* = 280) constituted the largest age group, accounting for 46.1% of the total sample population, followed by those aged 30–39 years (*n* = 240, 39.5%). Most of the nurses held a bachelor’s degree as the highest degree (*n* = 353, 58.2%), followed by those holding associate degrees (*n* = 218, 35.9%). Considering the years of service, 35.1% (*n* = 213) of the nurses had been employed in the hospital for more than 5 years and 27.8% (*n* = 169) had been employed for more than 1 year but less than 3 years (Table 3). In terms of the variable supervisor leadership, its scores varied with gender (*t* = −1.99, *p* < 0.05). With almost half of the personnel being under 30 years old, in terms of the variable policies and guidelines set by the supervisors, its scores varied with age (*F* = 3.14, *p* < 0.05). The more professional training the nurses received at school, the more demanding they may be of their supervisors. Thus, educational background affected the scores of the following items: the policies and guidelines set by the supervisors (*F* = 5.03, *p* < 0.01), educational training (*F* = 3.43, *p* < 0.05), shift schedules (*F* = 3.09, *p* < 0.05), salary and benefits (*F* = 3.11, *p* < 0.05), and work environments (*F* = 3.37, *p* < 0.05). Nurses with fewer years of service were less familiar with the organizational culture and the leadership behavior of the supervisors and needed more time to adapt. Thus, it was apparent that the years of service affected the scores of the following items: the policies and guidelines set by the supervisors (*F* = 6.75, *p* < 0.001), educational training (*F* = 5.62, *p* < 0.01), shift schedules (*F* = 11.60, *p* < 0.001), internal communication (*F* = 9.31, *p* < 0.001), salary and benefits (*F* = 5.00, *p* < 0.01), work environments (*F* = 13.42, *p* < 0.001), and supervisor leadership (*F* = 11.43, *p* < 0.001). 

### 3.2. Validation of the Relationships among the Latent Variables and Satisfaction with Supervisor Leadership 

Table 4 presents the overall revised model. Satisfaction with the policies and guidelines was positively associated with satisfaction with internal communication (B = 1.07, *p <* 0.001) (H1 was supported). Furthermore, the regression coefficient of the relationship between satisfaction with the policies and guidelines on satisfaction and the work environment was positive (B = 1.07, *p <* 0.001) (H2 was supported). The regression coefficient of the relationships between satisfaction with internal communication on satisfaction and supervisor leadership was positive (B = 0.40, *p <* 0.001) (H3 was supported). The regression coefficient of the relationship between satisfaction with the work environment on satisfaction and supervisor leadership was not significant (B = −0.40, *p* > 0.05) (H4 was not supported). The regression coefficient of the relationship between satisfaction with educational training on satisfaction and supervisor leadership was positive (B = 0.23, *p <* 0.01) (H5 was supported). The regression coefficient of the relationship between satisfaction with shift schedules on satisfaction and supervisor leadership was positive (B = 0.40, *p <* 0.001) (H6 was supported). The regression coefficient of the relationship between satisfaction with the salary and benefits as well as supervisor leadership was not significant (B = −0.09, *p* > 0.05) (H7 was not supported).

As shown in Table 4, satisfaction with the policies and guidelines was positively associated with internal communication (*B* = 0.92, *p* < 0.001). Moreover, satisfaction with internal communication had a direct relationship with satisfaction with supervisor leadership (*B* = 0.34, *p* < 0.001). These results indicate that the relationship of satisfaction with the policies and guidelines on satisfaction with supervisor leadership was associated with internal communication. Therefore, internal communication had a mediating effect on the relationship between the aforementioned variables.

### 3.3. The Finalized Structural Equation Model 

As shown in Table 5, H1: The regression coefficient between the policies and guidelines and work and communication was positive (B = 1.04, *p <* 0.001). H2: The regression coefficient between work and communication and leadership and operating performance was positive (B = 0.34, *p <* 0.001). H3: The regression coefficient between educational training and leadership and operating performance was positive (B = 0.24, *p <* 0.01). H4: The regression coefficient between the shift schedules and leadership and operating performance was positive (B = 0.34, *p <* 0.001). 

**Figure 2:**
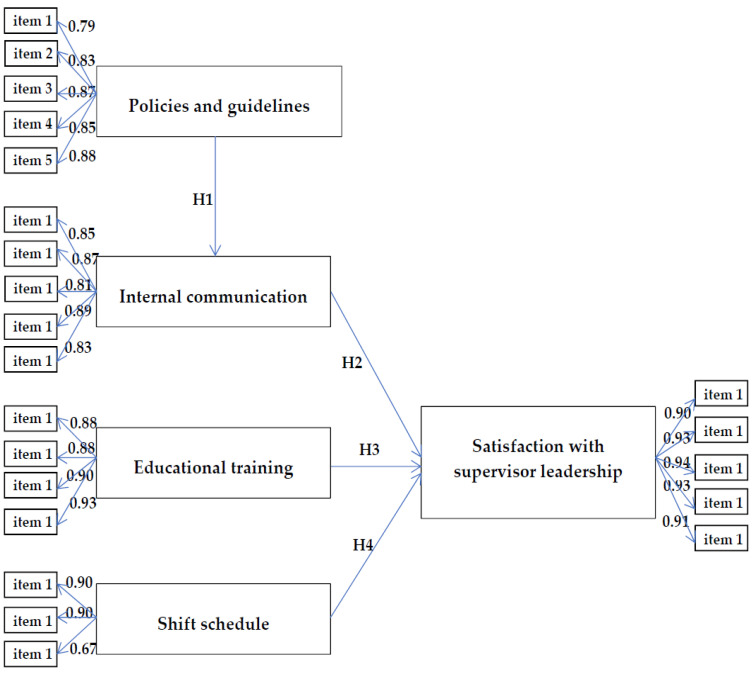
The finalized model reflects the relationships between various variables and nurses’ satisfaction with the supervisor leadership.

With regard to the hypothesized model in this study, routes that were not statistically significant were removed one by one, which made this model more complete. The finalized structural model was created, which reflects the relationships between the variables and nurses’ satisfaction with the supervisor leadership. This is illustrated in Figure 2.

## 4. Discussion

This scale revealed that nurse satisfaction with supervisor leadership was associated with shift schedules, internal communication, educational training, and policies and guidelines. Thus, this scale shows that nurses have different demands of their supervisors in different aspects, thereby demonstrating the applicability and feasibility of this scale in clinical practices. This study used social exchange theory to explore the nurses’ perspectives. SEM analysis confirmed that shift schedules, internal communication, and educational training are factors associated with overall satisfaction with supervisor leadership. Satisfaction with the policies and guidelines is indirectly associated with the overall satisfaction with supervisor leadership through internal communication. Specifically, internal communication is a mediator between satisfaction with the policies and guidelines and overall satisfaction with supervisor leadership.

When developing our questionnaire, we did not compare its criterion-related validity with that of MSQ, PES-NWI, or MMSS. However, in terms of reliability, the internal consistency of the question items ranged from 0.851 to 0.961, which indicates good reliability. In terms of validity, expert validity ranges from 0.7 to 1.0, indicating good content validity. Additionally, each aspect contained at least three question items, and their standardized factor loadings fell between 0.666 and 0.948, which means that this scale has good reliability and validity [26,27,28].

Our results revealing that the shift schedules, internal communication, and educational training were associated with overall satisfaction with supervisor leadership were similar to the findings reported by Wieke Noviyanti et al. and Hariyati and Safril [29,30]. These results indicate that for health care teams to be successful, they must have common goals with the hospital and engage in mutual support and communication within the organization in addition to having active leaders, a favorable work environment, and appropriate team training. This study found that the educational background of nurses was associated with their satisfaction with supervisor leadership, particularly in the aspects of policies and guidelines for supervisor leadership. As proposed by a study conducted by Yaktin et al., nurses with a higher education may have higher expectations of the supervisor leadership and tend to feel dissatisfied with their supervisors when the organization fails to meet their needs or expectations [31].

As social exchange theory emphasizes that reciprocity happens in social exchanges, workers want to achieve a work–life balance. Therefore, self-scheduling or a fixed shift schedule may increase the nurses’ job satisfaction [32,33]. Dall’Ora et al. stated that 12 h shifts are more favorable than a combination of 8- and 12-h shifts because a smaller number of shift handovers promotes teamwork [34]. Managers in the nursing department should encourage the cultivation of workplace culture and value the off-duty hours of nurses. Nurses should be adequately rested when they hand over their shifts and be allowed to refuse overtime [35,36]. 

Social exchange theory posits that when supervisors and employees develop a positive relationship, both parties tend to feel obligated to take action to benefit one another. Reciprocity will then be practiced in supervisor–employee relationships as well as in their job roles over time and in communication between the two sides [37]. This study revealed that nurses of different ages and with different lengths of service show different levels of satisfaction toward the leadership exercised by their supervisors. This is consistent with findings from Ma et al., who identified that because health organizations have lower expectations of younger nurses or nurses with shorter lengths of service, nurses who are long-term employees or are relatively older may feel unappreciated and exhibit dissatisfaction toward the leadership of their supervisors when they are not given job autonomy or opportunities to be involved in decision-making [38]. 

Leaders can design internal communication methods and formulate policies and operating procedures through organizational integration to reallocate resources and promote personnel performance [39]. Organizational integration involves leaders formulating policies and plans within the department to state personal needs and specific duties so that nurses can clearly receive messages from the organization and understand their job requirements, which consequently ensures that each individual works with others to achieve the organization’s objectives and plans [40]. Thus, leaders can create a satisfying organizational culture that prompts nurses to make efforts toward achieving common values. To boost morale and performance, leaders must transform from conventional commanding, controlling, and supervising roles to roles focusing on encouraging cooperative communication and learning motivation among nurses within the department [41].

Gender differences have been a major issue in society. Because men and women have different expectations of leadership and management, their satisfaction with supervisors also differs [42]. Of the nurses’ personal characteristics in this study, gender as a factor only affected the score of supervisor leadership. Note, however, that with only 17 males in this survey, there was only limited explanatory power to predict the relationship of gender on the participants’ satisfaction with supervisor leadership. 

Only such revolutionary leadership management can enhance the satisfaction of personnel with leaders and with their jobs and also make the personnel believe that their supervisors can create an environment with effective internal communication and maintain a pleasant work atmosphere [43]. Identical to the findings of this study, communication exerts partial mediating effects on the relationship between satisfaction with the policies and guidelines and satisfaction with supervisor leadership. Only after policies and guidelines are established can they be promoted through internal communication to enhance the degree of satisfaction that nurses feel toward the leadership of their supervisors.

As suggested by social exchange theory, employees develop more trust and job satisfaction when their supervisors arrange adequate training for them. Hence, educational training provided by supervisors can be the key to overall employee satisfaction [44]. Educational training should be adjusted based on feedback from the participating nurses. Fan et al. emphasized that knowledge flipping can be used to implement and enhance support for leadership, and that having nurses preview the difficult topics before classes can result in higher effectiveness [45]. Good leaders can use educational training to stimulate the creativity and problem-solving abilities of low-level personnel [46].

## 5. Study Limitations

This study surveyed nurses working in a single hospital and adopted purposeful sampling. The study results could have been influenced by the subjective judgment and bias of the participants, which could not be controlled; thus, it was difficult to control for sampling bias. Because the scale developed in this study was an initial effort in research, it is advisable that random sampling is used to further test the validity and reliability of the scale in order to explore its sensitivity and specificity as well as increase the applicability of this scale across other areas worldwide.

## 6. Conclusions 

The scale assessing nurse satisfaction with supervisor leadership developed in this study has proven to be valid and reliable as well as applicable in clinical settings. Following our preliminary results, it is advisable to increase the number of enrollments for further validation. Overall, the satisfaction of nurses with the policies and guidelines was directly associated with their satisfaction with supervisor leadership and also induced greater satisfaction with supervisor leadership through internal communication. Furthermore, satisfaction with shift schedules and educational training was directly associated with the overall satisfaction with supervisor leadership, and satisfaction with shift schedules and internal communication had a greater association with satisfaction with supervisor leadership. In contrast, satisfaction with the salary and benefits and the work environment was not significantly associated with the overall satisfaction with supervisor leadership. 

This means that in the nursing profession, the most important means of enhancing the satisfaction of nurses with supervisor leadership include establishing good shift schedules and diverse and effective channels of communication, improving training and opportunities for development, and formulating policies and guidelines that the nurses can accept. We therefore suggest that medical institutions offer courses on how to schedule shifts, communicate, arrange educational training, and formulate policies and guidelines to nurse supervisors to enhance their management capabilities and create a positive practice environment in which nurses are happy to work.

## Figures and Tables

**Table 1:** Overall goodness-of-fit assessment of the model.

Overall Fit Indices	Assessment Standard (*p*)	Value	Result
Absolute fit indices			
Likelihood ratio χ^2^	≥0.05	1928.82 *** (*df* = 392)	-
GFI	≥0.90	0.81	Acceptable
AGFI	≥0.90	0.77	Poor
SRMR	≤0.08	0.07	Good
RMSEA	≤0.08	0.08	Good
Incremental fit indices			
NFI	≥0.90	0.90	Good
NNFI	≥0.90	0.91	Good
RFI	≥0.90	0.89	Acceptable
IFI	≥0.90	0.92	Good
CFI	≥0.90	0.92	Good
Parsimony-adjusted indices			
PGFI	≥0.50	0.68	Good
PNFI	≥0.50	0.81	Good
PCFI	≥0.50	0.83	Good
Likelihood ratio χ^2^/*df*	≤3	4.92	Acceptable

Abbreviation: Goodness-of-fit index, *GFI*; Adjusted goodness-of-fit index, *AGFI;* Standardized root mean square residual, *SRMR;* root mean square error of approximation, *RMSEA*; Normed fit index, *NFI*; Non-normed fit index, *NNFI*; Relative fit index, *RFI;* Incremental fit index, *IFI;* Comparative fit index, *CFI;* Parsimonious goodness-of-fit index, *PGFI*; Non-normed fit index, *NNFI;* Parsimonious comparative fit index, *PCFI*. *** *p* < 0.001.

**Table 2:** Confirmatory factor analysis results for each variable.

Latent Variable	Manifest Variable	SFL	*t* Value	Individual Item Reliability (*R*^2^)	CR	AVE (%)
Policies and guidelines	Question item 1	0.78	-	0.606	0.92	0.70
	Question item 2	0.82	22.55 ***	0.678		
	Question item 3	0.86	23.92 ***	0.742		
	Question item 4	0.84	23.29 ***	0.712		
	Question item 5	0.87	24.17 ***	0.753		
Educational training	Question item 1	0.91	-	0.827	0.95	0.82
	Question item 2	0.90	35.98 ***	0.815		
	Question item 3	0.89	34.71 ***	0.793		
	Question item 4	0.92	37.51 ***	0.840		
Shift schedule	Question item 1	0.90	-	0.812	0.87	0.69
	Question item 2	0.90	31.40 ***	0.801		
	Question item 3	0.67	19.06 ***	0.443		
Internal communication	Question item 1	0.85	-	0.729	0.93	0.72
	Question item 2	0.87	28.34 ***	0.755		
	Question item 3	0.81	24.98 ***	0.653		
	Question item 4	0.89	29.57 ***	0.790		
	Question item 5	0.83	26.04 ***	0.686		
Salary and benefits	Question item 1	0.88	-	0.781	0.91	0.71
	Question item 2	0.88	29.77 ***	0.766		
	Question item 3	0.88	30.15 ***	0.776		
	Question item 4	0.73	21.57 ***	0.529		
Work environment	Question item 1	0.75	-	0.559	0.91	0.71
	Question item 2	0.78	19.75 ***	0.609		
	Question item 3	0.92	23.69 ***	0.844		
	Question item 4	0.92	23.59 ***	0.837		
Supervisor leadership	Question item 1	0.93	-	0.872	0.96	0.83
	Question item 2	0.95	46.82 ***	0.899		
	Question item 3	0.92	42.34 ***	0.852		
	Question item 4	0.84	31.89 ***	0.702		
	Question item 5	0.90	38.95 ***	0.811		

Abbreviation: Standardized factor loading, SFL; Construct reliability, CR; Average variance extracted, AVE. *** *p* < 0.001.

**Table 3:** Comparison of the nurses’ personal characteristics and scores of the latent variables in the questionnaire.

Item	Total	Policies and Guidelines	Educational Training	Shift Schedule	Internal Communication	Salary and Benefits	Work Environment	Supervisor Leadership
	*N* (%)	*M* (*SD*)	*M* (*SD*)	*M* (*SD*)	*M* (*SD*)	*M* (*SD*)	*M* (*SD*)	*M* (*SD*)
Gender								
Female	590 (97.2)	13.2 (2.6)	10.4 (2.2)	7.6 (1.8)	12.7 (2.8)	9.3 (2.6)	9.4 (2.2)	13.1 (2.9) *
Male	17 (2.8)	13.5 (2.6)	10.8 (2.0)	8.3 (1.5)	13.4 (2.7)	10.0 (2.2)	10.1 (2.7)	14.3 (2.5)
Age								
20–29 years old	280 (46.1)	13.1 (2.6) *	10.2 (2.2)	7.6 (1.8)	12.6 (2.8)	9.3 (2.6)	9.4 (2.7)	13.0 (3.0)
30–39 years old	240 (39.5)	13.1 (2.7)	10.4 (2.1)	7.6 (1.7)	12.8 (2.7)	9.2 (2.5)	9.3 (2.5)	13.1 (2.9)
40–49 years old	82 (13.5)	14.1 (2.3)	11.0 (2.0)	8.2 (1.8)	13.3 (2.8)	9.9 (2.5)	9.8 (2.5)	13.7 (2.6)
Above 50 years old	5 (0.9)	14.0 (2.6)	10.9 (2.5)	8.3 (1.7)	13.1 (3.0)	10.3 (2.8)	9.7 (2.8)	13.8 (2.6)
The highest degree or level of education								
Associate Degree	218 (35.9)	13.6 (2.6) **	10.6 (2.1) *	7.9 (1.7) *	13.0 (2.7)	9.6 (2.5) *	9.7 (2.6) *	13.4 (2.8)
Bachelor’s Degree	353 (58.2)	13.0 (2.6)	10.2 (2.2)	7.5 (1.8)	12.6 (2.8)	9.1 (2.6)	9.2 (2.6)	13.0 (2.9)
Master’s Degree	36 (5.9)	12.2 (2.7)	9.7 (2.3)	7.4 (1.9)	12.0 (2.8)	9.4 (2.5)	8.9 (2.3)	12.4 (2.9)
The years of service								
<1 year	98 (16.2)	13.8 (2.4) ***	10.9 (2.0) **	8.4 (1.4) ***	13.7 (2.4) ***	9.7 (2.4) **	10.4 (2.2) ***	14.3 (2.3) ***
≥1 year but <3 years	169 (27.8)	13.3 (2.5)	10.4 (2.1)	7.7 (1.7)	12.8 (2.7)	9.6 (2.4)	9.6 (2.5)	13.1 (2.9)
≥3 years but <5 years	127 (20.9)	12.4 (2.7)	9.8 (2.2)	7.1 (1.8)	11.9 (3.0)	8.6 (2.5)	8.5 (2.5)	12.2 (3.1)
≥5 years	213 (35.1)	13.2 (2.6)	10.3 (2.2)	7.5 (1.9)	12.6 (2.8)	9.2 (2.7)	9.1 (2.7)	12.9 (2.9)

Abbreviation: Mean, *M*; Standard deviation, *SD*. * *p* < 0.05, ** *p* < 0.01, *** *p* < 0.001.

**Table 4:** Relationship analysis of the structural equation model.

Hypothesis	Path Relationship	Non-Standardized Coefficients	*t* Value	Standardized Coefficients	Results
H1	Policies and guidelines→Internal communication	1.07	21.50 ***	0.92	Supported
H2	Policies and guidelines→Work environment	1.07	15.96 ***	0.76	Supported
H3	Internal communication→Satisfaction with supervisor leadership	0.40	5.90 ***	0.34	Supported
H4	Work environment→Satisfaction with supervisor leadership	−0.04	−0.99	−0.04	Not supported
H5	Educational training→Satisfaction with supervisor leadership	0.23	2.89 **	0.20	Supported
H6	Shift schedule→Satisfaction with supervisor leadership	0.40	6.91 ***	0.43	Supported
H7	Salary and benefits→Satisfaction with supervisor leadership	−0.09	−1.84	−0.09	Not supported

** *p* < 0.01, *** *p* < 0.001.

**Table 5:** Relationship analysis of the finalized structural equation model.

Hypothesis	Path Relationship	Non-standardized Coefficients	*t* Value	Standardized Coefficients	Results
H1	Policies and guidelines→Internal communication	1.04	21.29 ***	0.90	Supported
H2	Internal communication→Satisfaction with supervisor leadership	0.34	6.02 ***	0.30	Supported
H3	Educational training→Satisfaction with supervisor leadership	0.24	3.13 **	0.21	Supported
H4	Shift schedule→Satisfaction with supervisor leadership	0.34	6.60 ***	0.37	Supported

** *p* < 0.01, *** *p* < 0.001.

## Data Availability

The data analyzed during the current study are available from the corresponding author upon reasonable request.

## Supplementary Materials

The following supporting information can be downloaded at: https://www.mdpi.com/article/10.3390/ijerph20053933/s1, Supplementary File S1: Nurse Satisfaction with Supervisor Leadership Scale.
