# Supplementary material for: Factors Related to Nurse Satisfaction with Supervisor Leadership
Source: Int J Environ Res Public Health. 2023 Feb 22;20(5):3933. doi: 10.3390/ijerph20053933 (PMC10001521; doi:10.3390/ijerph20053933)
Supplement: Supplementary file 1 [file ijerph-20-03933-s001.zip › ijerph-2152030-supplementary.pdf]

Nurse Satisfaction with Supervisor Leadership Scale

Dear nurses:

Thank you for filling out this questionnaire. The purpose of this questionnaire is to understand your satisfaction with your supervisor's leadership at work. This is an anonymous questionnaire, and the results will only be used for academic analysis. Please make your most honest responses. We appreciate it.

Please provide your basic information.
What is your gender?
☐ Male
☐ Female

What is your age?
☐ 20-29 years old
☐ 30-39 years old
☐ 40-49 years old
☐ above 50 years old

What is your highest degree or level of education?
☐ Associate Degree
☐ Bachelor's Degree
☐ Master's Degree
☐ Doctoral Degree

What is the total years of service?
☐ less than 1 year
☐ more than 1 year but less than 3 years
☐ more than 3 years but less than 5 years
☐ more than 5 years

Please read the question items carefully and choose the number that best fits your own situation. You may only circle one response for each item.

**Item score**	**1**	**2**	**3**	**4**	**5**
**Satisfaction statements**	Dissatisfied	Slightly dissatisfied	Neutral	Slightly satisfied	Satisfied
**Policies and guidelines**					
1 Our hospital has propagated hospital-wide notices of our mission, prospects, and goals through various channels.					
2 I am ___ with the patient safety procedures that our hospital implements.					
3 I am familiar with the policies and guidelines of our hospital.					
4 Our hospital is committed to interacting with our community and actively participates in community events.					
5 Our hospital emphasizes teamwork and complete implementation.					
**Educational training**					

6	I am ___ with our hospital's employee education and training system.					
7	Our hospital encourages training opportunities for self-education and development.					
8	The department arranges the professional training courses that I need.					
9	The hospital provides the professional training courses that I need.					
**Shift schedules**						
10	Our hospital has various types of shift schedules.					
11	Shift scheduling is based on the personal factors and opinions of the employees.					
12	Employees can use the personnel system to query their own shifts and attendance data.					
**Internal communication**						
13	I am ___ with work-related communication with other departments.					
14	I am ___ with the scope of my duties and my role at work.					
15	Hospital information is distributed via the internet, an electronic bulletin board, or e-mail.					
16	Each department has clearly defined duties.					
17	I am ___ with the employee complaint channels of our hospital.					
**Salary and benefits**						
18	I am ___ with the content of my salary.					
19	I am ___ with our hospital's benefits.					
20	I am ___ with our hospital's mutual aid money program.					
21	I am ___ with our hospital's transportation measures.					
**Work environment**						
22	The environment of our hospital is clean.					
23	Our hospital is bright and well lit.					
24	The hardware facilities of our hospital are safe.					
25	Our hospital provides the tools and equipment required for our work.					
**Supervisor leadership**						
26	I am ___ with the attitude that my direct supervisor uses to treat those under his/her supervision.					
27	I am ___ with the way my direct supervisor leads.					
28	I am ___ with the praise given by my direct supervisor when we complete a task.					
29	I am ___ with the professional knowledge and capabilities of my direct supervisor.					
30	I am ___ with how my supervisors have managed our department.					
